# Whether low-dose metronomic oral cyclophosphamide improves the response to docetaxel in first-line treatment of non-triple-negative metastatic breast cancer

**DOI:** 10.18632/oncotarget.18539

**Published:** 2017-06-16

**Authors:** Jian Zhang, Leiping Wang, Zhonghua Wang, Biyun Wang, Jun Cao, Fangfang Lv, Sheng Zhang, Zhimin Shao, Xichun Hu

**Affiliations:** ^1^ Department of Medical Oncology, Fudan University Shanghai Cancer Center, Department of Oncology, Shanghai Medical College, Fudan University, Shanghai, China; ^2^ Department of Breast Surgery, Fudan University Shanghai Cancer Center, Department of Oncology, Shanghai Medical College, Fudan University, Shanghai, China

**Keywords:** metronomic chemotherapy, oral cyclophosphamide, docetaxel, metastatic breast cancer

## Abstract

Oral metronomic chemotherapy may target tumor cells indirectly via antiangiogenic activity, restoration of anticancer immune response, or induction of tumor dormancy. We initiated the single-center, randomized, open-label, phase II study to determine whether the addition of metronomic cyclophosphamide to docetaxel (T) (w/o trastuzumab) improves overall response rate (ORR) as first-line treatment among patients with non-triple-negative metastatic breast cancer (MBC). Eligible patients with previously untreated non-triple-negative MBC were randomly assigned (1:1) to receive 3-weekly cycles of Metro-TC (T 75mg/m^2^, d1 plus oral cyclophosphamide 50 mg daily) or T alone. All patients received treatment until disease progression, unacceptable toxicity, or withdrawal of consent. The primary endpoint was ORR. Finally, 35 patients were randomized to Metro-TC group while 31 to T group. Median treatment cycles of T for both groups were 8. ORR was not improved by addition of metronomic cyclophosphamide to T (71.4% vs. 51.6%; *P* = 0.09). There was no statistically significant difference with regard to progression free survival (median 18.5 vs. 11.7 months; *P* = 0.07) or overall survival (median 33.7 vs. 33.6 months; *P* = 0.84) between the two group. Grade 3/4 adverse events (eg. neutropenia [100% vs. 100%], febrile neutropenia [29% vs. 29%], and neurotoxicity [6% vs. 3%]) were also comparable. There were no treatment-related deaths. We conclude that concomitant administration of metronomic cyclophosphamide and T does not appear to be a significantly active schedule for first-line treatment of non-triple-negative MBC.

## INTRODUCTION

Metastatic breast cancer (MBC) is essentially an incurable disease and the prognosis has changed little over the past decade with median overall survival of patients is still only 2–3 years [1–3]. Consequently, treatment goals are to optimize both length and quality of life [1].

Metronomic chemotherapy, refers to treatment at regular, close intervals without prolonged breaks at doses significantly less than the maximum-tolerated dose [4]. This treatment modality may target tumor cells indirectly via inhibiting angiogenesis and vasculogenesis by continuously exposing the more slowly proliferating tumor endothelial cells to cytotoxic therapy [5–8]. In addition, a chronic administration has an immunomodulatory effect that leads to additional mechanism for its antitumor effect [9–11]. Low dose metronomic chemotherapy may offer several advantages, including low toxicity and treatment irrespective of the resistance profile of the tumor cell population [12–14]. Methotrexate, cyclophosphamide, capecitabine, and taxanes are the most common metronomic chemotherapy anticarcinogenic drugs [15]. Metronomic schedule of cyclophosphamide is effective in multiple tumor types, including ovarian cancer, [16, 17] prostate cancer, [18, 19] breast cancer, [6, 20–23] some refractory solid tumors, [24] and lymphomas [24, 25].

Docetaxel is one of the most active chemotherapeutic drugs against breast cancer. Phase III randomized trials of single-agent docetaxel yielded promising results in patients with MBC [26, 27]. As first-line treatment phase III randomized studies have demonstrated that for HER2-negative MBC, single-agent docetaxel achieved overall response rate (ORR) of 37.9%–46.4%, median progression free survival (PFS) of 8.2 months and overall survival (OS) of 27.2–31.0 months; [28, 29] for HER2-positive MBC, docetaxel plus trastuzumab was shown to be ORR of 59.3%-72%, median PFS or time to progression (TTP) of 11.1–12.4 months and OS of 35.7–40.8 months [30–32]. Thus, with significant activity and manageable toxicity, docetaxel monotherapy is considered as one of standard chemotherapies in first-line treatment of MBC.

Based on the preclinical evidence that metronomic chemotherapy may target tumor cells indirectly via antiangiogenic activity, restoration of anticancer immune response, or induction of tumor dormancy, and previously observed encouraging activity with minimal toxicity, we initiated the single-center, randomized, open-label, phase II study (NCT01526499) to determine whether the addition of low-dose metronomic oral cyclophosphamide to docetaxel (with or without trastuzumab) improves ORR compared with docetaxel alone as first-line treatment among patients with non-triple-negative MBC.

## MATERIALS AND METHODS

### Study Population

Patients included in the study were required to meet the following criteria: at least 18 years of age; histologically confirmed non-triple-negative breast cancer with metastatic disease; measurable disease according to Response Evaluation Criteria in Solid Tumors (RECIST) 1.1 criteria; a life expectancy of no less than 3 months; Eastern Cooperative Oncology Group (ECOG) performance status of 0 to 1; absolute neutrophil count (ANC) ≥ 1.5 × 10^9^/L; platelet count ≥ 75 × 10^9^/L; hemoglobin ≥ 9 g /dL; total serum bilirubin ≤ 1.5 × upper limit of normal (ULN); AST/ALT ≤ 2.5 × ULN (≤ 5 × ULN in case of liver metastases); serum creatinine ≤ 1.0 × ULN (calculated creatinine clearance ≥ 50 mL/min). All patients with no prior chemotherapy, endocrine therapy, trastuzumab for metastatic disease were allowed. Prior taxane-based adjuvant or neoadjuvant chemotherapy was permitted if relapse had occurred ≥ 6 months after the discontinuation of taxane-based therapy; prior trastuzumab adjuvant or neoadjuvant therapy (without prior taxane therapy) was permitted if ≥ 6 months had relapsed since the end of such therapy; prior taxane and trastuzumab-based adjuvant or neoadjuvant therapy was permitted if ≥ 12 months had relapsed since the end of such therapy. Patients with central nervous system metastases or who were pregnant were ineligible.

The study was approved by the Fudan University Cancer Hospital Ethic Committee for Clinical Investigation (approval number: 1111104–11). The study was carried out in accordance with the Declaration of Helsinki. Written informed consent was obtained from all patients prior to enrollment.

### Randomization and masking

We randomly assigned eligible patients (1:1) to receive either docetaxel + metronomic cyclophosphamide (Metro-TC) or docetaxel (T) alone. Simple randomization was done, with no stratification factors, via an interactive web-response system. After investigators completed the random assignment forms and checked the inclusion criteria, the allocated treatment was determined by the study coordinator through the system. Patients, investigators, and outcome assessors were aware of treatment group assignment.

### Treatment

Patients were randomly assigned in a 1:1 ratio to receive 3-weekly cycles of Metro-TC (docetaxel 75 mg/m2, day 1 plus continuous oral cyclophosphamide 50 mg daily) or T alone (75 mg/m2, day 1). Patients with HER2-overexpressed tumors should receive trastuzumab with a loading dose consisting of 8 mg/kg over 90 minutes and 6 mg/kg over 60 minutes every 3 weeks. All patients received treatment until disease progression, unacceptable toxicity, or withdrawal of consent. Per protocol, up to two dose reductions of docetaxel per patient were allowed from 75 mg/m^2^ to 60 mg/m^2^ then to 45 mg/m^2^ when necessary. Treatment was terminated when patients met grade 3/4 peripheral neuropathy or grade 4 hypersensitivity. In Metro-TC group, if docetaxel was discontinuated owing to docetaxel-specified toxicities, metronomic cyclophosphamide could be continued until disease progression, unacceptable toxicity, or withdrawal of consent. Maintenance endocrine therapy after discontinuation of docetaxel treatment but before documented disease progression was not prohibited for HR positive patients.

### Assessment

Pretreatment evaluations included a detailed medical history, physical examination, laboratory evaluation, and performance status. Laboratory evaluation included a routine blood count, biochemistry including electrolytes, renal and liver function tests, and urinalysis. Adverse events (AEs) and concomitant medications were recorded at the end of each cycle throughout the study period until 30 days after the last dose of a study treatment was administered. Toxicity was evaluated and graded according to National Cancer Institute Common Terminology Criteria for Adverse Events, version 4.0.

Radiographic scans (CT scan or MRI) for efficacy evaluation were conducted at baseline and every two treatment cycles thereafter per RECIST 1.1. The best response was documented. For patients without disease progression at the end of treatment, radiographic assessment was performed every 2 months within the first 6 months and every 3 months thereafter until documented progression or death. Survival status was assessed approximately every 3 months.

### Statistical methods

The primary endpoint was ORR in the intention-to-treat (ITT) population. ITT analysis is a comparison of the treatment groups that includes all patients as originally allocated after randomization. According to a two-sided test with an alpha-error of 0.05 and a beta-error of 0.30, and assuming a 40% ORR for the T arm and an expected an ORR in experimental arm of 70%, a sample size of 62 patients (31 for each arm) is required. The secondary endpoints were PFS and OS. PFS was *defined* as the interval between randomization date and documented disease progression, or death as a result of any cause in patients with no evidence of disease progression. OS was defined as the interval between randomization date and death. Safety issues including incidence and severity of AEs were also investigated. For the safety analysis, we assessed data for all patients who received at least one dose of study treatment.

All statistical analyses were carried out using SPSS 20.0 (SPSS, Inc.). Between-treatment comparisons of the ORR and frequencies of AEs on treatment were performed using the χ2 test. PFS and OS were estimated and 95% confidence intervals were calculated by means of the Kaplan–Meier method. All P values and confidence intervals reported are two-sided, and all analyses are of data for the ITT population unless otherwise noted. Univariate survival curves were generated by the Kaplan-Meier method. All tests were two-sided and P values < 0.05 were considered statistically significant.

## RESULTS

### Patients

**Figure 1:**
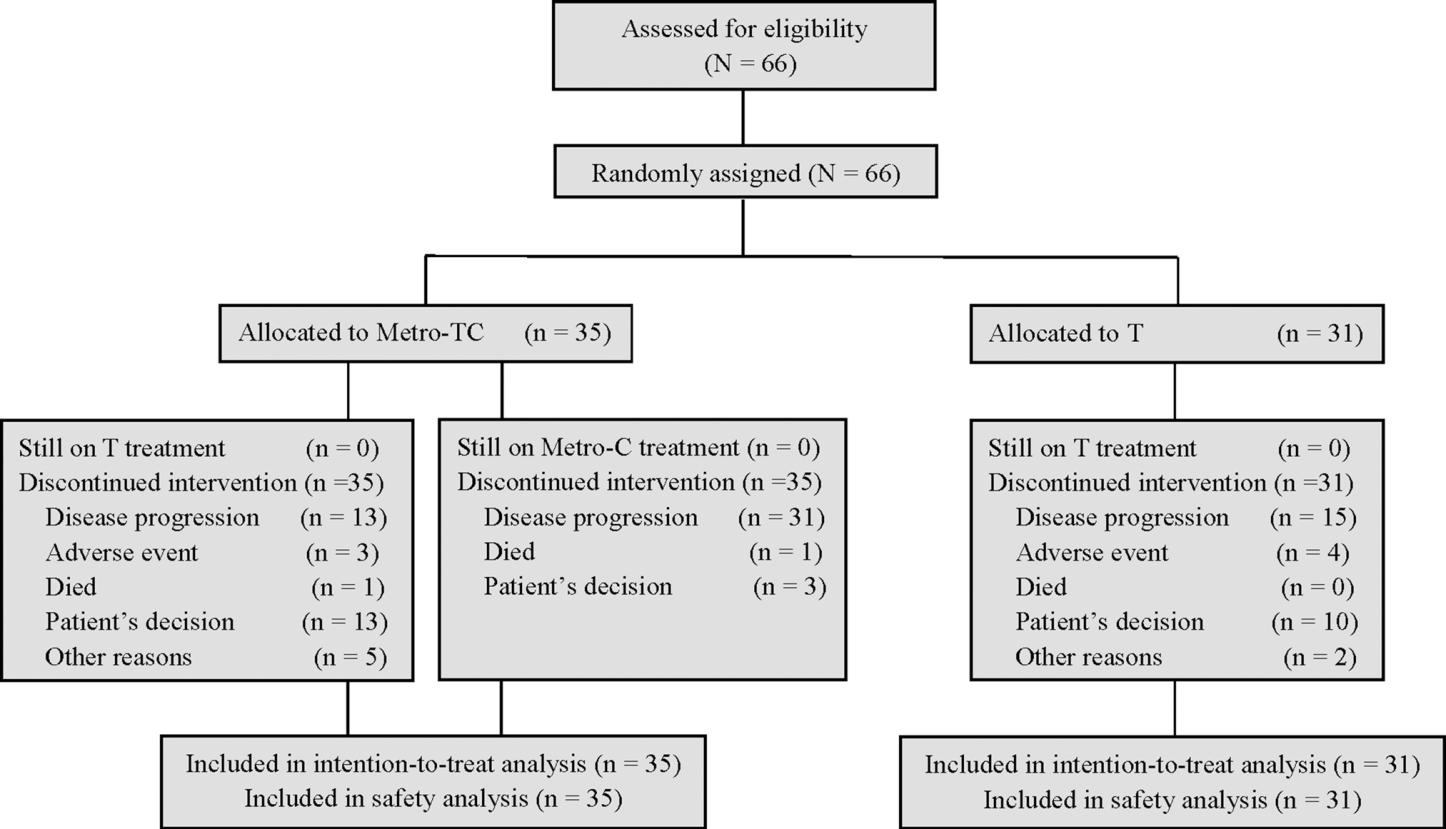
Trial profile

Between Dec 2011 and Nov 2012, 66 patients with non-triple-negative MBC were recruited, in which 35 patients were randomized to Metro-TC group while 31 to T group (Figure 1). The baseline characteristics of the patients were well balanced in the two treatment groups (Table 1). The majority of the patients (83%) were hormonal receptor (HR) positive; 32% were HER2 over-expressed; 85% had visceral metastasis; 49% had involvement of more than two organs; and 45% were disease-free interval (DFI) ≤ 2 years. The majority of the patients (73%) had been exposed to adjuvant chemotherapy, with 36% having received taxane-based therapies.

**Table 1:** Demographics and clinical characteristics of all randomly assigned patients

Characteristics	Metro-TC (*n* = 35)	T (*n* = 31)
Age, years		
Mean ± SD	52.3 ± 5.6	53.6 ± 4.3
Range	31–63	40–64
HR and HER2 status		
HR+ and HER2 negative	24 (68.6)	21 (67.7)
HR+ and HER2 positive	6 (17.1)	4 (13.0)
HR- and HER2 positive	5 (14.3)	6 (19.3)
Metastatic sites		
Liver	16 (45.7)	13 (41.9)
Lung	23 (65.7)	18 (58.1)
Bone	19 (54.3)	13 (41.9)
Soft tissue	25 (71.4)	19 (61.3)
Visceral	31 (88.6)	25 (80.6)
Non-visceral only	5 (14.2)	4 (12.9)
No. of metastatic sites		
1–2	18 (51.4)	16 (51.6)
≥ 3	17 (48.6)	15 (48.4)
DFI, months		
*de novo* stage IV	8 (22.9)	9 (29.0)
DFI ≤ 2 years	9 (25.7)	4 (12.9)
DFI > 2 years	18 (51.4)	18 (58.1)
Prior therapy		
Adjuvant Chemotherapy	26 (74.3)	22 (71.0)
Anthracycline	23 (65.8)	22 (71.0)
Taxane	14 (40.0)	10 (32.3)
Adjuvant hormonal therapy	25 (71.4)	22 (71.0)
Adjuvant Trastuzumab	0	0

Abbreviation: Metro-TC, docetaxel + metronomic cyclophosphamide ; T, docetaxel; HR, hormonal receptor; DFI, disease-free interval.

Data are presented as *n* (%) unless otherwise stated.

### Efficacy

At the time of analysis, with median follow-up time of 40 months, 65 patients (34 patients in Metro-TC group; 31 in T group) had experienced disease progression, whereas 34 patients (18 patients in Metro-TC group; 16 in T group) had died.

**Figure 2:**
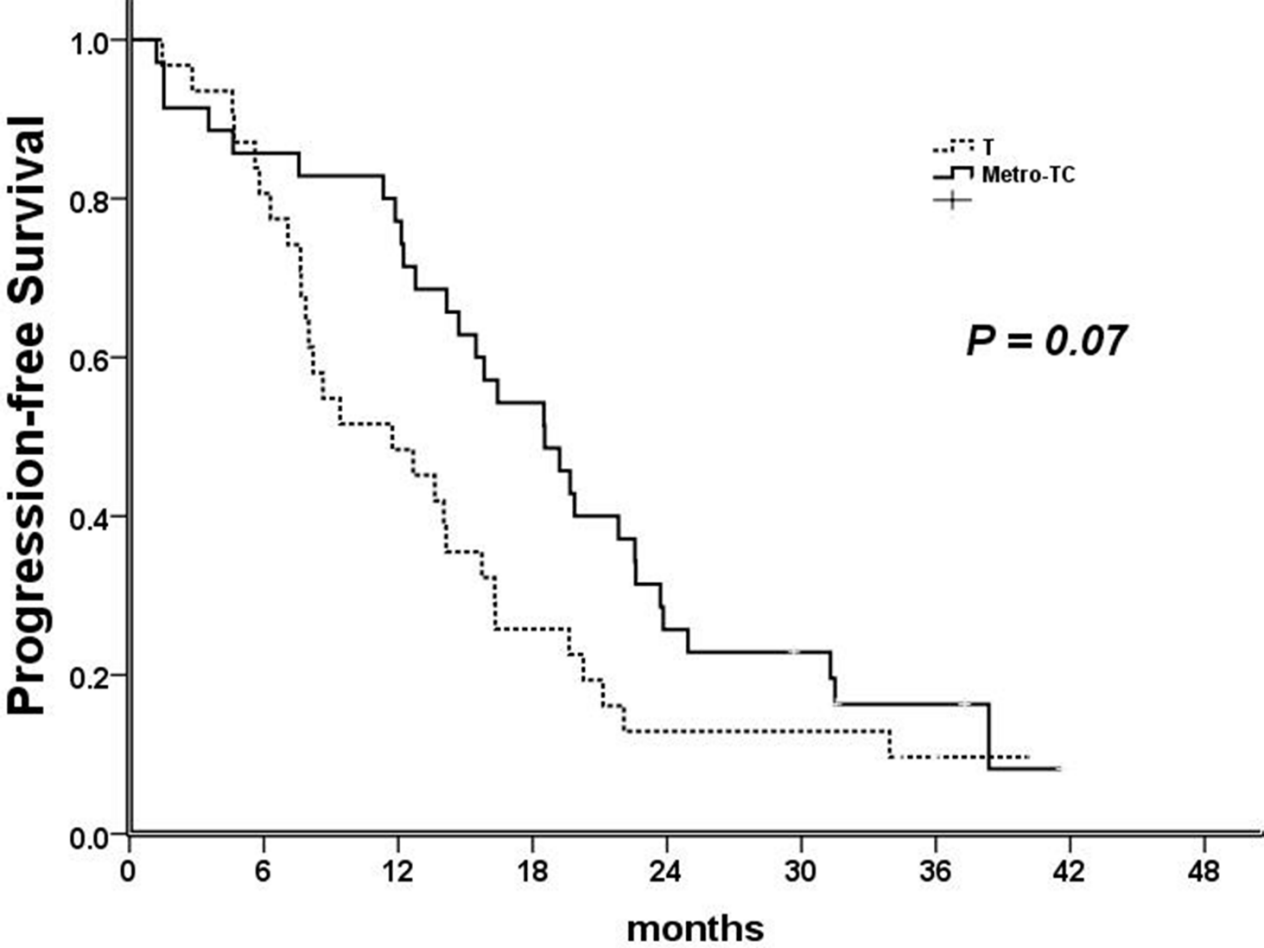
Kaplan-Meier estimates of progression-free survival (PFS) for the ITT population

Overall efficacy was shown in Table 2. Overall response rate (ORR) were 71.4% (25/35) in Metro-TC and 51.6% (16/31) in T group, respectively (*P* =.09). All the responses took place during the period of docetaxel-containing treatment. In intention-to-treat population, median PFS was 18.5 months (95% CI, 14.1 to 23.0) for the Metro-TC group and 11.7 months (95% CI, 5.8 to 17.7) for the T group (Figure 2). The difference in PFS between the two treatment groups was not statistically significant (hazard ratio, 1.596; 95% CI, 0.948 to 2.687) (*P* =.07). The median OS was 33.7 months (95% CI, 27.3 to 40.0) in Metro-TC group and 33.6 months (95% CI, 25.3 to 41.9) in T group. The difference in OS between the two treatment groups was not statistically significant (*P* = .84).

**Table 2:** Summary of efficacy analyses, ITT population

	Metro-TC (*n* = 35)	T (*n* = 31)	*P*
Response, *n* (%)			
Overall response	25 (71.4%)	16 (51.6%)	0.09 (chi-square)
Complete response	1 (2.9%)	1 (3.2%)	
Partial response	24 (68.6%)	15 (48.4%)	
Stable disease	7 (20.0%)	11 (35.5%)	
Progressive disease	2 (5.7%)	2 (6.4%)	
Not assessable	1 (2.8%)	2 (6.5%)	
PFS, months			
Median (95% CI)	18.5 (14.1 - 23.0)	11.7 (5.8 - 17.7)	0.07 (log-rank)
PFS, subgroup			
HER2 positive*	19.7 (18.2 - 21.1)	14.0 (4.0 - 24.1)	0.26 (log-rank)
HER2 negative	15.8 (11.2 - 20.4)	9.4 (4.1 - 14.7)	
OS, months			
Median (95% CI)	33.7 (27.3 - 40.0)	33.6 (25.3 - 41.9)	0.84 (log-rank)

Abbreviation: Metro-TC, docetaxel + metronomic cyclophosphamide ; T, docetaxel.

*All HER2-positive patients received trastuzumab.

### Toxicity

Table 3 depicted drug-related toxicities observed. Grade 3/4 adverse events (eg. neutropenia [100% vs. 100%], febrile neutropenia [29% vs. 29%], and neurotoxicity [6% vs. 3%]) were comparable between the two groups. AEs were mainly docetaxel-related and grade 3/4 ones with high frequencies (≥ 5%) included neutropenia (100%) and febrile neutropenia (29%). The only significant difference between the two treatment groups was grade 1–4 mucositis (10% in T vs. 43% in Metro-TC). Serious adverse events were seen in 4 patients (3 with febrile neutropenia and 1 with severe diarrhea leading to prolonged hospitalization) in Metro-TC group and 3 (with febrile neutropenia leading to prolonged hospitalization) in T group. There were no treatment-related deaths. Reasons for discontinuation of docetaxel in Metro-TC and T groups were as follows: disease progression in 28 patients (13 vs. 15), AEs in 7 (3 vs. 4, see Table 3), death for non-tumor causes in 1 (1 vs. 0), patient’s decision in 23 (13 vs. 10), and other reasons in 7 (5 vs. 2). No episodes of febrile neutropenia were observed in either treatment group after discontinuation of docetaxel. Reasons for discontinuation of cyclophosphamide in Metro-TC group were disease progression in 31 patients, death for non-tumor causes in 1, and patient’s decision in 3.

**Table 3:** Drug related adverse events*

Adverse Event	Metro-TC (*n* = 35)		T (*n* = 31)	
	Grade 1–4	Grade 3/4	Grade 1–4	Grade 3/4
Any event	35 (100%)	35 (100%)	31 (100%)	31 (100%)
Hematologic				
Neutropenia	35 (100%)	35 (100%)	31 (100%)	31 (100%)
Febrile neutropenia	10 (29%)	10 (29%)	9 (29%)	9 (29%)
Anemia	22 (63%)	−	19 (61%)	−
Thrombocytopenia	2 (6%)	−	3 (10%)	−
Non-hematologic				
Peripheral neurotoxicity	20 (57%)	2 (6%)**	17 (55%)	1 (3%)**
Peripheral edema	19 (54%)	1 (3%)**	12 (39%)	1 (3%)**
Nausea	19 (54%)	−	11 (36%)	−
Nail changes	18 (51%)	−	17 (55%)	1 (3%)
Alopecia	17 (49%)	−	15 (49%)	−
Fatigue	16 (46%)	1 (3%)	14 (45%)	1 (3%)
Vomiting	16 (46%)	−	5 (16%)	
Mucositis	15 (43%)	−	3 (10%)	1 (3%)**
Arthralgia	15 (43%)	−	11 (36%)	−
Rash	5 (14%)		7 (23%)	
Diarrhea	5 (14%)	1 (3%)**	5 (16%)	−

Abbreviation: Metro-TC, docetaxel + metronomic cyclophosphamide ; T, docetaxel.

Data are presented as n(%) unless otherwise stated.

*Listed are all grades of adverse events that are possibly, probably, or definitely related to the treatment with an incidence of 5% or more in either group or of grade 3 or above with an incidence of 2% or higher in either group.

**Adverse events leading to discontinuation of docetaxel.

### Treatment delivered

Patients received the protocol-specified continuous oral cyclophosphamide 50mg daily and docetaxel in Metro-TC treatment group. The median metronomic oral cyclophosphamide administered was 555 days (range, 12 to 972) were administered. Exposure to docetaxel was comparable between both treatment groups. The median number of cycles administered was 8 (range, 1–14) for Metro-TC group and also 8 (range, 1–19) for T group. Sixty-three percent of patients in Metro-TC and 61% in T completed 8 cycles. The median dose intensity of docetaxel was 22.0 mg/m^2^ per week in Metro-TC group and 21.4 mg/m^2^ per week in T group. Both proportion of reductions and delays were comparable between the two groups (Table 4).

**Table 4:** Treatment exposure

	Metro-TC (*n* = 35)	T (*n* = 31)
Metronomic cyclophosphamide (days)	555 (12–972)	NA
Docetaxel		
Median number of cycles (range)	8 (1–14)	8 (1–19)
Dose intensity, mg/m^2^ per week (range)	22.0 (18.8–25)	21.4 (16.4–25)
Number of dose reduction to ≤ 60 mg/m^2^ (proportion)	10 (29%)	9 (29%)
Proportion of cycles delayed or interrupted	9.4%	10.7%

Abbreviation: Metro-TC, docetaxel + metronomic cyclophosphamide ; T, docetaxel.

Twenty-one (70%) and 16 (64%) HR positive patients received maintenance endocrine drugs in Metro-TC and T groups after discontinuation of docetaxel treatment but before documented disease progression, respectively. The majority (87%) of maintenance endocrine therapies were aromatase inhibitors w/o luteinizing hormone-releasing hormone agonists.

## DISCUSSION

Metronomic chemotherapy has emerged as an effective treatment with a major clinical advantage: it minimizes the toxic side effects of the drugs employed, thereby allowing their safe and long term administration [20]. In the current study, we reported results from NCT01526499, a prospective, open label, randomized, phase II trial aiming to evaluate the efficacy and safety of metronomic administration of oral cyclophosphamide in addition to docetaxel for first-line treatment of non-triple-negative MBC. The primary endpoint was not met. The addition of metronomic oral cyclophosphamide to T did not result in a statistically significant improvement in term of ORR.

Three important points of metronomic schedule should be noted. First, whether other mechanisms, such as tumor dormancy, are also involved in the effectiveness of metronomic administration of chemotherapy or if it is superior to conventional maximum tolerated dose chemotherapy remains questionable, which is required to be determined on larger patient groups in randomized controlled phase III trials such as NCT01131195 comparing the efficacy and safety of body surface area-based dosing paclitaxel and bevacizumab versus fixed dosing metronomic cyclophosphamide and capecitabine and bevacizumab as first-line therapy in advanced breast cancer patients [33]. Second, whether the potential preclinical superiority has a successful clinical translation. Actually, a randomized phase II neoadjuvant trial of adding low-dose daily oral cyclophosphamide to letrozole did not increase ORR in 114 elderly ER-positive breast cancer patients [21]. Third, the identification of patients who might benefit more from metronomic schedule is crucial for the optimization of the treatment strategy. Up to date, no well-defined markers, including VEGF, sVEGFR1, and sVEGFR2, to predict the response to metronomic chemotherapy have been identified [6, 20, 23].

For MBC, the first trial to assess the efficacy of metronomic chemotherapy was reported by Colleoni et al. and in this phase II study using daily oral cyclophosphamide 50 mg/day continuously and methotrexate 2.5 mg bid twice-weekly, among the 63 evaluable patients, the ORR arrived at 19% and clinical benefit rate (complete response + partial response + stable disease >24 weeks) was 31.7% with minimal toxicity [33] Subsequent studies confirmed the efficacy of metronomic cyclophosphamide and methotrexate, [20, 23, 34] or in combination with trastuzumab in patients with HER2 positive MBC [35]. In a phase II trial, heavily pretreated MBC patients received continuous metronomic capecitabine 1500 mg once a day, among 58 assessable patients, the clinical benefit rate was 62%, median TTP and OS were 7 and 17 months, respectively [36]. Dellapasqua et al. evaluated metronomic oral capecitabine 1500 mg/day and cyclophosphamide 50 mg/day plus bevacizumab in patients who had received no more than three previous regimens of chemotherapy for advanced disease. In 46 assessable patients, median TTP was 10.5 months, ORR and clinical benefit rates were 48% and 68%, respectively [22]. These studies thus offered a potentially new strategy for treating MBC with metronomic low-dose chemotherapy. It should be noted that all of the above trials were not randomized controlled ones. However, our study presented no benefit of response when adding metronomic oral cyclophosphamide to first line docetaxel among women with non-triple-negative MBC in a randomized controlled way.

In fact, the value of metronomic schedule of chemo drugs was widely tested not only in the metastatic but in the adjuvant setting. The recently reported IBCSG 22–00 adjuvant trial showed the addition of cyclophosphamide and methotrexate maintenance (CMM) to standard chemotherapy resulted in 4.1% absolute reduction in 5-year DFS for triple-negative disease [37]. Ongoing trials including the phase III SYSUCC-001 (NCT01112826) trial will clarify the value of adding metronomic approach to conventional therapies in patients with triple-negative breast cancer [38].

The most frequent (≥ 5%) grade 3/4 AEs in both groups of our study were neutropenia (100%) and febrile neutropenia (29%) and no significant differences between treatments were noted. The tolerability profile of docetaxel and Metro-TC was as expected in our study, and when compared with the scientific literature, no new safety concerns were identified. A phase III trial comparing three doses (60, 75, and 100 mg/m^2^) of docetaxel for second-line treatment of advanced breast cancer showed that grade 3/4 neutropenia occurred in 76.4%, 83.7%, and 93.4% and febrile neutropenia occurred in 4.7%, 7.4%, and 14.1% of patients, respectively [39]. While in first line HERNATA study, significantly more grade 3/4 febrile neutropenia (36.0%), infection (25.1%) and fever (4.3%) were reported with docetaxel 100 mg/m^2^ [31]. Patients in Metro-TC group had a higher incidence of grade 1–4 mucositis, which, however, generally self-limiting and well managed.

Our study has several limitations. First, the non-triple-negative breast cancer could be divided into several subgroups and different molecular subtypes might lead to clinical heterogeneity. Second, maintenance endocrine therapy was not prohibited for HR positive patients in our study, which might compromise the benefit of metronomic cyclophosphamide. However, treatments exposed to endocrine maintenance therapy were generally balanced between Metro-TC and T groups. Last, our study demonstrated a non-statistically but marginally significant improvement in term of ORR (71.4% vs. 51.6%; *P* = 0.09) or PFS (18.5 vs. 11.7 months; *P* = 0.07) with the addition of metronomic oral cyclophosphamide. Actually, if there is an effect which is almost within significance level, increasing the sample size may maximize the chance of uncovering a specific difference, which is also statistically significant. Further well-designed large-scale trials are required to elucidate the issue.

In conclusion, although no additional toxicities were observed, concomitant administration of metronomic oral cyclophosphamide and docetaxel does not appear to be a significantly active schedule for first-line treatment of non-triple-negative MBC.
